# Heme and HO-1 Inhibition of HCV, HBV, and HIV

**DOI:** 10.3389/fphar.2012.00129

**Published:** 2012-10-04

**Authors:** Warren N. Schmidt, M. Meleah Mathahs, Zhaowen Zhu

**Affiliations:** ^1^Department of Internal Medicine and Research Service, Veterans Affairs Medical Center, University of IowaIowa City, IA, USA; ^2^Department of Internal Medicine, Roy G. and Lucille A. Carver College of Medicine, University of IowaIowa City, IA, USA

**Keywords:** Heme, viruses, HCV, HIV, HBV, metalloporphyrins, biliverdin, proteases

## Abstract

Hepatitis C virus, human immunodeficiency virus, and hepatitis B virus are chronic viral infections that cause considerable morbidity and mortality throughout the world. In the decades following the identification and sequencing of these viruses, *in vitro* experiments demonstrated that heme oxygenase-1, its oxidative products, and related compounds of the heme oxygenase system inhibit replication of all 3 viruses. The purpose of this review is to critically evaluate and summarize the seminal studies that described and characterized this remarkable behavior. It will also discuss more recent work that discovered the antiviral mechanisms and target sites of these unique antiviral agents. In spite of the fact that these viruses are diverse pathogens with quite profound differences in structure and life cycle, it is significant that heme and related compounds show striking similarity for viral target sites across all three species. Collectively, these findings strongly indicate that we should move forward and develop heme and related tetrapyrroles into versatile antiviral agents that could be used therapeutically in patients with single or multiple viral infections.

## Introduction

Hepatitis C virus (HCV), Hepatitis B virus (HBV), and Human immunodeficiency virus (HIV) are three of the most common chronic viral infections worldwide. All of these viruses share common risk factors and modes of transmission including sexual, human blood product transfusion, and intravenous drug use (Koziel and Peters, 2007). In the decades following discovery and sequencing of all three viruses, there has been a constant medical need to update treatment regimens and employ new and more versatile antiviral agents. This review will focus on *in vitro* and molecular studies that have evaluated metalloporphyrins, specifically heme, and related derivatives, for their virucidal activity against the three viruses. What are emerging from these collective works are not only fascinating pictures of multiple viral targets to explain the antiviral activities of these metalloporphyrins, but there is also a promise that these compounds can be developed successfully into powerful, yet versatile antiviral agents.

## The Heme and Heme Oxygenase Systems

**Figure 1:**
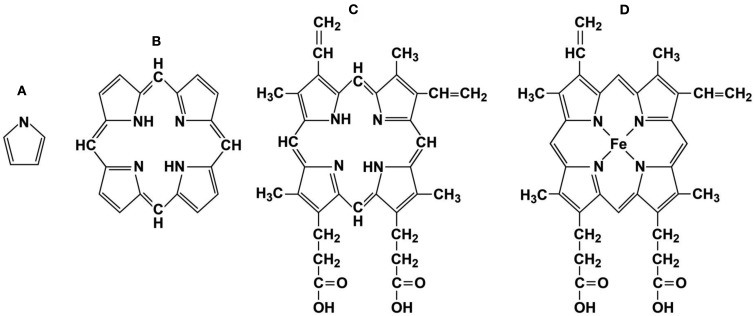
**Representative porphyrin structures**. **(A)** pyrrole ring, **(B)** porphyrin ring, **(C)** protoporphyrin IX, and **(D)** Heme, iron protoporphyrin IX.

Heme (iron protoporphyrin IX) is the most common metalloporphyrin (MP) in eukaryotic cells and is essential for life. Heme is constructed from a highly conserved sequence of eight enzymatic reactions that sequentially link precursors into four pyrrole precursor rings to form the tetrapyrrole, protoporphyrin IX. In the final anabolic step, ferrous iron is inserted by the enzyme ferrochelatase to form heme or iron protoporphyrin IX (Figure 1; Heinemann et al., 2008). Heme is regularly complexed into respiratory proteins such as hemoglobin and myoglobin to form a vital oxygen carrying and delivery platform. As a prosthetic group heme also performs essential activities for electron transfer and oxidation (Gray and Winkler, 1996). Because of the critical nature of all these basic activities, it is not surprising that virtually all steps of heme synthesis and degradation are tightly regulated with oxidative balance and many signaling pathways (Mense and Zhang, 2006).

Since the 1970s, commercial preparations of heme (*Hemin* and *Panhematin*), have been approved by the FDA for use in patients with acute porphyria. In addition to iron, natural metalloprotoporphyrins (MPP) and a variety of synthetic MP can complex other metals such as zinc, copper, cobalt, magnesium, manganese, tin, nickel, or chromium. Naturally occurring MPs serve as necessary co-factors for numerous oxygenases, peroxidases, and catalases. ZnPP, formed naturally during times of iron deficiency, is an excellent serum marker for iron deficiency anemia (Labbe, 1992) and has generated therapeutic interest for the treatment of jaundice of the newborn.

**Figure 2:**
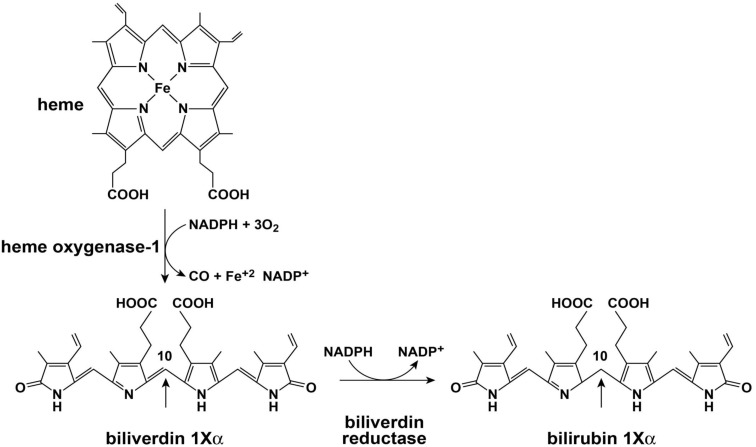
**Heme oxygenase and biliverdin reductase enzymes**.

Initial catalysis of heme occurs via oxidation with heme oxidase (HO; Figure 2). The major isoforms of HO are HO-1 and HO-2. The role of HO-3 is not clear and is considered a pseudo transcript of HO-2 (Cruse and Maines, 1988; McCoubrey et al., 1997; Hayashi et al., 2004). HO-1 and HO-2 perform similar net enzymatic functions, yet only HO-1 is usually induced in response to cellular stressors such as hypoxia, cytotoxic agents, and infection (Immenschuh and Ramadori, 2000). HO opens the porphyrin ring of heme which is rate limiting for heme catabolism, and liberates equimolar ratios of Fe^+2^, carbon monoxide, and the linear tetrapyrrole biliverdin (BV). The reaction uses 3 mol of oxygen and reducing equivalents from NADPH: cytochrome P-450 (cytochrome *c*) reductase. BV is then rapidly converted to bilirubin (BR) by BV reductase (BVR). The entire sequence of heme oxidation and BV reduction likely takes place in synchrony upon a large complex of enzymes and co-factors that include HO, BVR, and NADPH (Ryter et al., 2006). BR and other tetrapyrrole derivatives are ubiquitous in nature and have a wide variety of functions in animals (McDonagh, 2001). Both HO-1 and BVR are highly inducible enzymes and respond to signaling and feedback in numerous cellular systems (Ryter et al., 2006; Kapitulnik and Maines, 2009). HO-1 induction provides important protection from oxidative stress and cellular damage (Abraham et al., 1995; Lee et al., 1996; Guo et al., 2001). Over the last two decades it is also becoming apparent that the precursors and catabolic products of the heme oxygenase system are capable of antimicrobial and antiviral activities. Of importance here, HO-1 induction or overexpression promotes a wide range of antiviral activities for the major viral pathogens discussed in this review: HIV, HBV, and HCV (Devadas and Dhawan, 2006; Protzer et al., 2007; Shan et al., 2007; Zhu et al., 2008).

## General Considerations of Virucidal Activities of Heme and Related Compounds

Heme and structurally related MPs have actually generated interest as antimicrobials and antiviral agents for some time (Stojiljkovic et al., 2001). Porphyrins induce antiviral reactions through both non-specific and specific interactions with either host or virus which typically interfere with the viral life cycle. Typical non-specific interactions include activities such as hydrophobic binding of the porphyrin with host cell membranes or viral envelope that inhibit viral binding and cellular entry. Generalized antiviral activities reflect the diversity of porphyrin structure as well as chemical complexity and versatility. Photoactive MPs absorb photons and partition into membranes to where they can exert a virucidal effect, especially with enveloped viruses as noted below.

In contrast, specific interactions of natural MPs appear highly dependent on structure and reflect the fact that the primary function of heme and similar MPs usually behave as crucial co-factors in specific oxidase and peroxidase reactions. Consequently, an emerging pattern suggests that natural MPs show more affinity and specificity for viral inhibition because they are the preferred cofactor for established cellular reactions that are metabolically protective for cellular stress which may also severely reduce viral fitness. This assertion is clearly tentative, however, and has not been formally studied.

Recently, a group of alkylated porphyrins was reported to inhibit hepadnaviruses, flaviviruses, filoviruses, and arena viruses (Guo et al., 2011). The antiviral effects of these compounds, or chlorophyllides (an alkylated porphyrin containing copper charged at neutral ph), were discovered by screening over 2,000 compounds for their ability to decrease HBV DNA in cultured cells. A derivative of chlorophyllide, the metal free chlorin e6a,was the most active compound of this group. Antiviral activity for other viruses such as HCV, HIV-1, Dengue (DENV-2), Marburg virus (MARV), Junin viruses (JUNV), and herpes simplex (HSV-1) was also shown. Activity had high EC_50_s in the low micromolar and nanomolar range for some viruses such as DENV and was selective for enveloped viruses. Non-enveloped viruses such as encephalomyocarditis (EMCV) and adenovirus (Ad) were highly resistant. The active porphyrin compounds caused disruption and dissociation of viral envelope proteins as assessed in a particle gel assay. These studies suggest use for these compounds as generalized virucidal agents for enveloped viruses.

Alternatively, an interesting antiviral application of some photoactive MPs has been studied for non-enveloped viruses such as Hepatitis A virus (HAV). HAV is a small picornavirus that is orally transmitted from fecal contaminated water and food. Like all picornaviruses (the family includes notable human pathogens such as polioviruses, enteroviruses, and rhinoviruses) the non-envelope structure of HAV presents challenges for prevention of infection because of its small size and resistance to inactivation. These viruses are highly resistant to common bacterial filtration techniques and the protein capsid renders the virus more resistant to chemical disinfection (Lemon et al., 1994). Casteel et al. (2004) showed that synthetic porphyrins could photo-inactivate HAV in plasma and other body fluids. Consequently, synthetic porphyrins may offer an effective and relatively safe approach to disinfection of non-enveloped viruses in various types of aqueous media. Potentially, this remarkable property could widely influence the epidemiology and pathogenesis of a variety of common pathogens that cause considerable morbidity and mortality worldwide.

Song et al. (1997) assessed the anti-HIV properties of various water-soluble polysulfonated and polycarboxylated porphyrins and their metal (Mn, Fe, and Ni) derivatives against HIV-1, HIV-2, and simian immunodeficiency virus infection in MOLT4 target cells. Some of these compounds were potent inhibitors of HIV infection and they likely interfere with the binding of HIV protein gp120 to the CD4 cellular receptor. Specific antibody studies demonstrated that the porphyrins directly bound to the gp 120 protein to inhibit formation and uptake of gp 120 and CD4+ T cell receptor binding complex. Interestingly, the gp 120 protein was also the probable binding site of a group of boronated porphyrins and other related MPs that were reported to bind gp120 and inhibit infection of CD4+ T cell line MT-2 (Debnath et al., 1994, 1999).

The antiviral activities of some more specialized synthetic porphyrins and derivatives against HCV have also been studied recently (Cheng et al., 2010). These workers evaluated structure-activity relationships of synthetic tetraphenylporphyrins and their anti-HCV properties as a proof-of- concept model for the development of proteomimetics in HCV drug discovery. Nanomolar levels of a biphenyl porphyrin derivative were noted to be the most potent inhibitor of full length Con1 replicons (Blight et al., 2002) but were less active for genotype 2a (JFH-1) subgenomic replicons (Kato et al., 2003). Furthermore, the porphyrin showed synergistic antiviral activity when incubated with replicons together with a known HCV protease inhibitor BILN 2061 as well as the antiviral cytokine α-interferon-2a. While the cellular targets for the porphyrin derivatives are not yet clear, these studies demonstrate that the tetraphenylporphyrin backbone is a useful scaffold on which to hang a variety of chemical groups and define structure-function relationships for additional antiviral development.

## Direct Effects of Heme and Related Compounds

Direct interactions of heme and related MPs are important medically for the development of new and improved antiviral agents against HIV, HBV, and HCV in addition to other human pathogens that cause persistent infection and chronic disease. Viral targets might include any of the key steps in a virus’ life cycle such as entry, replication, assembly, and exit from the host cell. Alternatively, interactions of MP that enhance pathogen recognition and immune response pathways would be expected to decrease viral fitness and replication mechanisms. In this regard, heme and related natural compounds have recently been shown to possess astonishingly specific antiviral protease and polymerase activities for both human hepatotrophic and HIV viruses. As discussed below, an emerging pattern demonstrates novel actions of heme that influence not only viral replication, but immune recognition, inflammation, primary interferon responses, apoptosis, and oxidative defense mechanisms.

## Actions on Specific Viruses

### Hepatitis C virus

Hepatitis C virus is a small enveloped RNA virus and occupies a single genus of Hepacivirus of the Flaviviridae family. This family also includes such human pathogens as yellow fever and dengue. HCV has a plus-stranded RNA genome with a single long open-reading frame that is translated into a large polyprotein, then processed by host and viral proteases (Lindenbach and Rice, 2005). The non-structural (NS) proteins NS3/4A protease, NS5B RNA dependent RNA polymerase (RdRp) and the NS5A protein, necessary for replication, have been the targets of intense research efforts to develop new antiviral drugs (Soriano et al., 2009). The ability to study the HCV life cycle and develop anti-HCV agents was initially hampered for nearly a decade by the inability to grow and pass the virus in cell culture or small animal models. However, viral replicon models began to appear by 1999 (Lohmann et al., 1999; Pietschmann et al., 2001; Blight et al., 2002) and these constructs enabled high throughput *in vitro* testing for a wide variety of potential antiviral drugs. In addition to replicons, defined infectious strains (Cai et al., 2005; Wakita et al., 2005) and patient wild type viruses (Castet et al., 2002; Gondeau et al., 2009) have been passed in primary hepatocytes and other permissive cell lines. Recently, reliable chimeric mouse-human models for *in vivo* testing of antiviral activity have also been introduced (Bissig et al., 2007, 2010; de Jong et al., 2010; Washburn et al., 2011).

## Direct Antiviral Effects of Heme and Derivatives on HCV

Indications that heme and its enzymatic products may interact with HCV actually arose from assays of human liver slices collected from HCV infected patients. Abdalla et al. (2004) evaluated relative expression of oxidative defense enzymes in infected patient liver samples as compared to uninfected control liver. HO-1 was dramatically reduced in HCV infected liver, in contrast to other antioxidative enzymes, catalase, CuZn and Mn super oxide dismutases which all remained unchanged. Furthermore, immunohistochemistry confirmed that the reduction of HO-1 expression was limited to hepatocytes, the site of HCV replication, and not Kupffer cells. Since chronic HCV infection causes hepatic inflammation and progressive fibrosis, HO-1 downregulation was quite unexpected as it might be anticipated that the enzyme would be induced as in other liver diseases (Makino et al., 2001; Bauer et al., 2003). Moreover, in the same study, autoimmune hepatitis and chronic HBV showed marked HO-1 upregulation in affected hepatocytes (Abdalla et al., 2004). These findings suggested that the virus can specifically modulate HO-1 expression although reasons for this behavior were not apparent at the time. Nevertheless, reduction of this important oxidative defense enzyme in the hepatocyte may indirectly contribute to progressive oxidative injury and facilitate fibrosis. In fact, later experiments demonstrated that overexpression of HO-1 in replicon cells promoted increased resistance to oxidant-induced cytotoxicity (Zhu et al., 2008).

Ensuing work by a number of groups has revealed dramatic innate anti-hepatitis C activities for heme and the oxidative products of the heme oxygenase system. It was first demonstrated that iron can inhibit the HCV NS5B RdRp by high affinity (Kd = −6 μM) binding to the divalent cation binding pocket of the polymerase (Fillebeen et al., 2005). The Kd for ferrous ion was found to be 500 times greater than the preferred Mg^+^ ion. Either divalent Mg or Mn is absolutely required for NS5B enzymatic activity (Ferrari et al., 1999; Bougie et al., 2003; Benzaghou et al., 2004). Antiviral activity of free iron has been confirmed by others (Yuasa et al., 2006; Zhu et al., 2008). However, free iron also induces HO-1 suggesting that the virucidal activity of Fe is likely more complicated than just binding and inactivating the polymerase (Hou et al., 2009). Iron would also be an unlikely choice as a therapeutic agent for HCV since it is usually considered to be a hepatotoxin (Ryter and Tyrrell, 2000) and it seems doubtful whether sufficient intracellular levels of free iron might be achieved *in vivo* to be useful therapeutically without causing cellular injury. Mild iron accumulation in HCV infected human liver samples has been correlated with more severe liver disease in some but not all studies (Beinker et al., 1996; Kayali et al., 2005, 2007). Some but not all earlier clinical studies with α-interferon treatment regimens suggested that phlebotomy could improve viral clearance and increase sustained virological responses to α-interferon (Di Bisceglie et al., 2000; Fontana et al., 2000; Desai et al., 2008). Finally, some of the experiments looking at the effects of iron on replication *in vitro* were actually performed using hemin to load cells with iron instead of iron salts (Fillebeen et al., 2005, 2007). Consequently the potency of free iron may have been overestimated since more recent studies have shown that heme as well as its initial oxidation product biliverdin can also directly inhibit the HCV NS3/4A protease (Zhu et al., 2010a).

**Figure 3:**
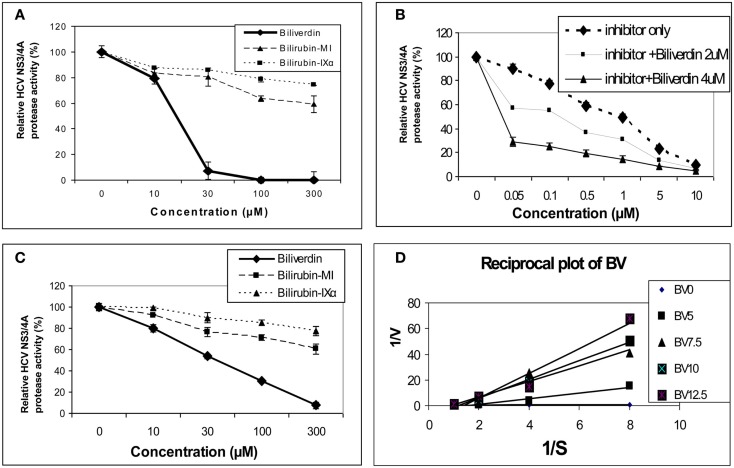
**Tetrapyrrole inhibition of HCV NS3/4A protease**. **(A,B)** Protease activity was determined fluorometrically (FRET assay) using recombinant NS3/4A enzyme and various concentrations of inhibitors. **(C)** Endogenous NS3/4A protease activity in microsomes of replicons was measured using the same FRET assay but employing endogenous, partially purified NS3/4A protease from replicon cells (Zhu et al., 2010a). **(D)** Reciprocal (Lineweaver–Burk) plot of substrate concentration vs. enzyme activity. Recombinant protease activity was determined fluorometrically. Each point is the mean ± SEM of 3–5 determinations per point. Plot of [BV] vs. either 1/Vap or Km/V (not pictured) showed highly significant linearity (*r* = 0.975 and *r* = 0.979 respectively, *p* < 0.005) indicating mixed inhibition of NS3/4A protease by BV (${K^{\prime }}_{\text{i}}$ = 1.1  mM and *K*_i_ = 0.6 mM, respectively). *0* to the commercial Inhibitor = NS3/4A protease competitive inhibitor, *AnaSpec* #25346. Biliverdin = >99% Biliverdin IX-α. Bilirubin-mixed isomers (MI) = 93% Bilirubin IX-α, and 6% associated Bilirubin isomers. Bilirubin IX-α = >99% bilirubin IX-α. (With permission Zhu et al., 2010a see original manuscript for further details.)

As noted above, earlier work demonstrated that BV and bilirubin (BR) were able to inhibit the aspartyl protease of HIV (McPhee et al., 1996). In contrast, HCV NS3/4A protease is serine activated with a classical common catalytic mechanism like other members of this large class of proteolytic enzymes (Love et al., 1996; Yan et al., 1998). Inhibition of serine activated proteases with a tetrapyrrole appears common as a recent study demonstrated that both BV and BR were capable of inactivating intestinal trypsin and chymotrypsin (Qin, 2007). In this regard, Zhu et al. (2010a) reported that BV, heme, and to a much lesser extent BR were able to attenuate HCV replication in NS and full length replicons *in vitro*. BV was found to be a potent inhibitor of recombinant NS3/4A protease using Fluorescence Resonance Energy Transfer (FRET) inhibition assays in contrast to BR (Figure 3). After testing a number of biologically relevant linear tetrapyrroles, BV showed the lowest IC_50_ of all compounds tested (9.3 μM) and was considerably more potent than its reduction product, BR (Figure 3A). Assays conducted in the presence of both BV and *AnaSpec* #25346, a known commercially available inhibitor, showed an additive effect. Lineweaver–Burk plots indicated a mixed competitive and non-competitive inhibitory mechanism (*K*_i_ = 0.6 μM and ${K^{\prime }}_{\text{i}}$ = 1.1 μM. Taken together, the kinetic experiments suggest there is associated non-competitive protease inhibition that probably occurs in an allosteric fashion and requires further study.

The marked difference in the avidity of BV and BR for the HCV NS3/4A protease may be explained by differences in structure and physical chemistries of the two linear tetrapyrroles. BV and BR have widely different secondary structures [see McDonagh, 2001 for review] in spite of the fact that they differ only by a double vs. single C–C bond at the 10 “hinge” position respectively (Figure 2). The hinge double bond restrains BV into a flat rather fixed limited plane. In contrast, the free rotation about the hinge single bond of BR allows internal hydrogen bonding of the COOH groups which folds the molecule into a hydrophobic “ridge–tile” structure (Nogales and Lightner, 1995). The NS3/4A protease has an unusually long and shallow active site groove that is quite atypical for serine activated proteases (Love et al., 1996). Presumably, the active site accommodates the flat BV molecule to allow stable occupation while the bulky hydrophobic BR fits poorly. Presently, it is unclear which functional groups interact with the serine active site triad and where potential allosteric sites may be located. However, these interactions appear important for future drug design of tetrapyrroles as antiviral agents.

**Figure 4:**
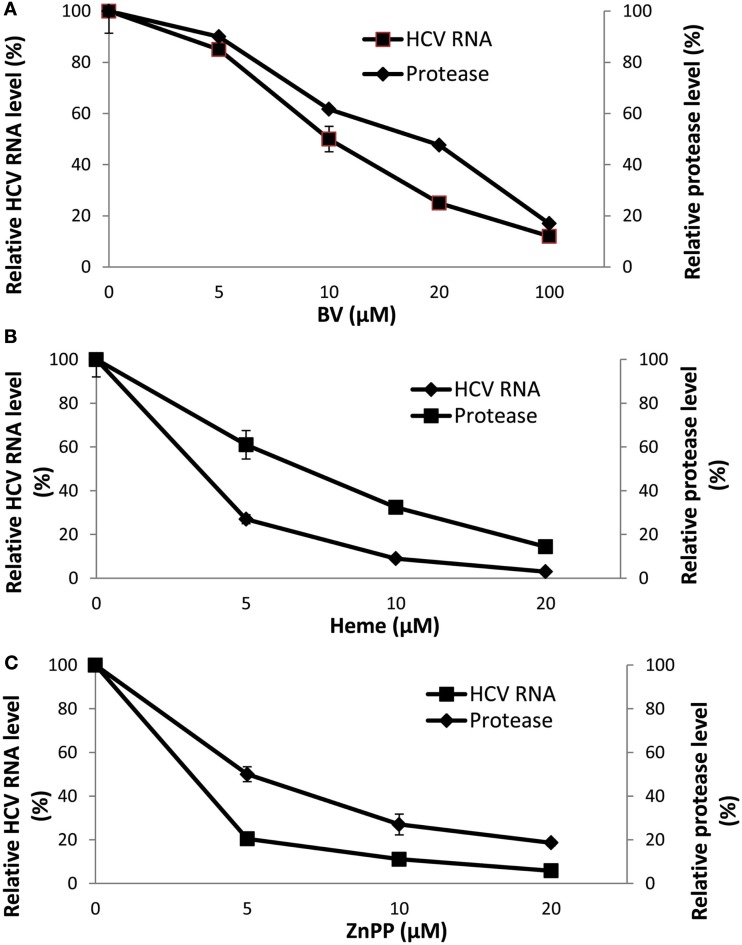
**Disappearance of HCV RNA and HCV NS3/4A inhibition with biliverdin, heme, or ZnPP. (A–C)** HCV RNA was determined in total RNA extracted from replicon cells incubated with the indicated concentrations of tetrapyrrole for 48 h. Independent determinations of the inhibitory activity of each tetrapyrrole for recombinant HCV NS3/4A was determined by FRET analysis (Zhu et al., 2010a). Results are means ± SEM of six determinations per point. (With permission; Zhu et al., 2011.)

At first we attributed the antiviral activity of heme to its induction of HO-1 and rapid oxidation with liberation of free iron and BV. However, direct testing of heme against the recombinant HCV NS3/4A protease in the FRET assay has revealed that heme, like BV, is also a direct protease inhibitor. Furthermore, some related metalloprotoporphyrins (MPP) such as ZnPP, also showed potent antiprotease and antiviral activities *in vitro* which occurred at quite similar inhibitor concentrations (Figure 4; Zhu et al., 2011). Further studies looking at relationships between tetrapyrrole structure, HCV protease binding affinity, and antiviral activity are ongoing in our laboratory. However, as noted above, planar compounds such as metal protoporphyrins appear to bind protease tightly in contrast to folded structures such as bilirubin derivatives, conjugated bilirubins, and mesoporphyrins (Zhu et al., 2010a).

Porphyrins with structures related to heme and ZnPP, such as the meso derivative ZnMP, have also been shown to have antiviral actions through HO-1 induction, however, with a more indirect mechanism. Transcriptional activation of HO-1 depends, in part, on Bach 1 a negative repressor of the HO-1 promoter region (Ogawa et al., 2001; Kitamuro et al., 2003). ZnMP was shown to induce the degradation of Bach 1 protein through increased proteasome degradation thus alleviating HO-1 promoter repression, facilitating HO-1 induction, and subsequent reduction of HCV RNA in NS replicons (Hou et al., 2008). Furthermore, a follow up study from the same group demonstrated that ZnMP could target HCV NS proteins such as NS5A for proteasomal degradation through ubiquitination (Hou et al., 2010).

## Indirect Anti-HCV Effects of Heme and Derivatives

Other reasons for the antiviral activity of heme and related molecules have also been investigated. Lehmann et al. (2010) reported that BV induced type I interferon expression in replicon cells which was accompanied by expression of interferon stimulated gene products. While this study was the first to describe direct interaction of an important natural tetrapyrrole with the innate immune system, the mechanism is not yet clear (Lehmann et al., 2010). Primary induction of interferon by BV *in vitro* has not been reported previously and appears contrary to the known anti-inflammatory character of BV and HO-1 induction. BV has been shown to induce nuclear accumulation of biliverdin reductase which then decreases nuclear NFkB in Hek293 cells (Gibbs and Maines, 2007; Gibbs et al., 2010). The latter findings are consistent with an immunosuppressive role for BV (Ollinger et al., 2007) in contrast to the more pro-inflammatory properties of heme (Gozzelino et al., 2010). On the other hand, in other cell types such as human myeloid cells, HO-1 induction was noted to be essential for IRF3 and NFkB activation and nuclear accumulation which are prerequisites for type I interferon induction (Tzima et al., 2009; Kalliolias and Ivashkiv, 2010). HO-1 knockout mice demonstrated that HO-1 induction is necessary for early phase activation of the innate immune sensing pathways TRIF–IRF3 and RIG-I–IRF3 with Sendai virus for the production of β-interferon and other primary response cytokines (Tzima et al., 2009; Koliaraki and Kollias, 2011). It is not known whether an HO-1 enzymatic product or a secondary mediator is required for these activities or whether they occur in other cell types such as hepatocytes. Nevertheless, these findings expand the influences of HO-1 into innate immunity and potentially other related pathways.

## BV and Heme Can Reverse HCV NS3/4A Inhibition of Type I Interferon Induction

**Figure 5:**
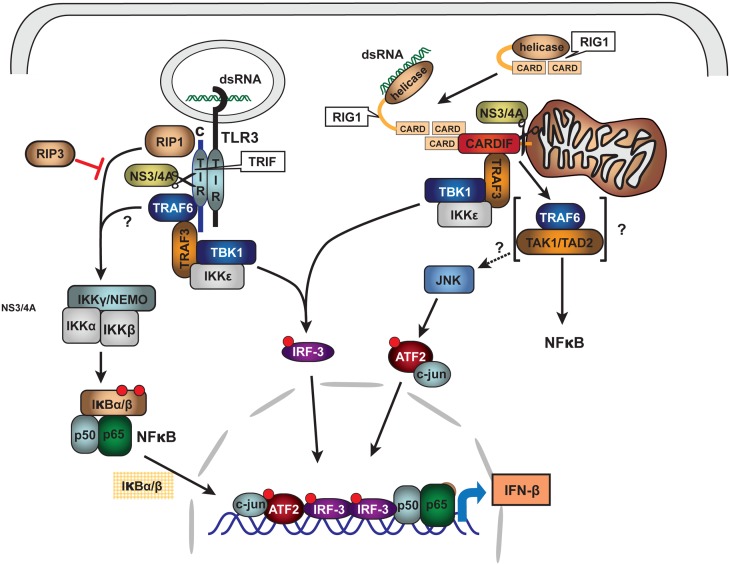
**Schematic of HCV NS3/4A protease inhibition of innate immune signaling pathways: TLR3 and RIG1**. Double stranded RNA (dsRNA) viruses or viruses with dsRNA intermediate activate innate immune system signaling pathways for type I interferon induction through binding to Pattern Recognition Receptors (PRR). For HCV, PRR are associated with at least two major signaling pathways: TLR3 and RIG1. Upon intracellular binding of dsRNA to associated adapter proteins TRIF and CARDIFF, respectively, signaling is transmitted through complex intermediate steps of recognition, binding, and phosphorylation leading to activation of the transcriptional factors IRF3 and NFκB. The activated nuclear transcription factors bind to type I interferon promoters and induce α/β-interferon transcription. HCV NS3/4A protease is known to cleave both TRIF and CARDIFF adapters thus crippling innate immune antigen recognition and signaling for type I interferon induction. With permission (Bode et al., 2007), see original review for terminology and abbreviations.

As is typical of viral pathogens, the NS3/4A protease has extra viral proteolytic activities that serve to increase the efficiency of viral infection and evasion of host immune system. NS3/4A can cleave pattern recognition receptors (PRR) or their interface adapter proteins within type I interferon signaling pathways and thus disable transcriptional induction of α and β interferon (Li et al., 2005b; Meylan et al., 2005; Loo et al., 2006; Bode et al., 2007; Kalliolias and Ivashkiv, 2010). The two best studied induction pathways likely important for HCV responses are those that recognize intracellular double stranded (ds) RNA and include the Toll like receptor 3 (TLR3) and the Retinoic acid inducible gene-1 (RIG1) pathways. HCV NS3/4A cleaves specific adapter proteins of both pathways including TRIF of the TLR3 system and Cardiff of the RIG1 system (Figure 5; Li et al., 2005b; Meylan et al., 2005; Bode et al., 2007). Knowing that heme and other tetrapyrroles inhibit the HCV protease, we recently investigated whether these compounds would restore type I interferon induction after disruption of interferon signaling with the protease.

**Figure 6:**
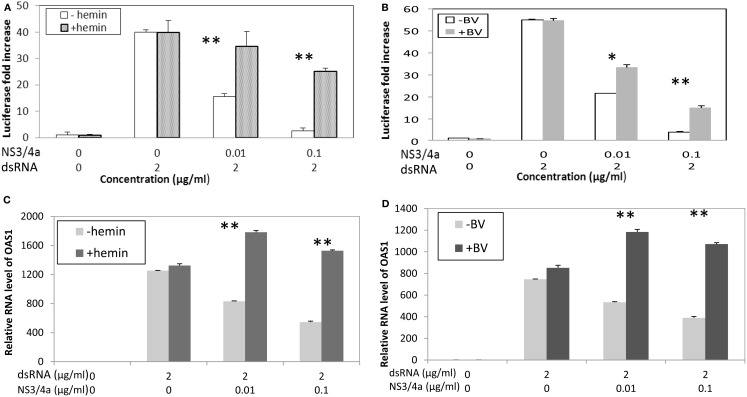
**Biliverdin and Heme can restore IFN promoter activation and Interferon Stimulated Response Genes (ISRG) after interference of IFN signaling with HCV NS3/4A. (A,B)** HEK 293 cells were transfected with dsRNA and vectors containing Type I IFN promoter-luciferase construct and complete NS3/4A sequence plasmid construct or control plasmid. Cells were then incubated with heme (20 μM) or Biliverdin (50 μM) for 48 h. Cellular lysates were prepared and assayed for interferon promoter activation using luciferase assay (Zhu et al., 2010b). Each point is the mean of six determinations (two separate cell culture incubations, and then three replicates for luciferase assay per incubation). **(C,D)** Parallel experiments showing that heme and BV can restore interferon stimulated response genes (ISRG) after inhibition with NS3/4A protease. HEK 293 cells were transfected with dsRNA with or without HCV NS3/4A plasmid then incubated with heme or BV for same time and concentrations as **(A,B)** OAS-1 mRNA was measured using *Real Time* RT-PCR. Each point is the mean of six determinations as described for **(A,B)** **p < 0.01; *p < 0.05; NS, not significant. (With permission Zhu et al., 2011.)

Clonal cell lines of Huh 7 such as Huh7.5 and Huh 515 are known to have poor dsRNA and dsDNA innate immunity recognition which is a likely reason that they are permissive for HCV replication (Li et al., 2005a; Cheng et al., 2007). Consequently, to evaluate the effects of heme on type I interferon signaling, it was necessary to construct a reconstituted model system. Hek293 cells were chosen because they are known to have relatively intact innate immune recognition and high transfection efficiency (Khvalevsky et al., 2007). We transfected dsRNA antigen into Hek293 cells and 48 h later assessed interferon induction using luciferase reporter gene constructs containing promoter regions for β interferon. Transfection of dsRNA elicited robust induction of type I interferon, however, this was clearly attenuated when cells were co-transfected with NS3/4A expression vectors (Figure 6). In contrast, incubation with heme or BV significantly restored induction in the presence of protease and depending on protease and substrate concentrations, this was nearly complete in some cases (Figures 6A,B; Zhu et al., 2011). Additionally, restoration of interferon induction in the presence of protease was sharply accompanied by induction of interferon stimulated response gene (ISRG) products such as OAS-1 as shown here (Figures 6C,D; Zhu et al., 2011). Furthermore, we noted that BV or heme only controls without protease, showed little ability to augment type I interferon induction when incubated with cells either with or without dsRNA antigen. This suggests that in the present system the tetrapyrroles are unlikely to induce type I interferons as a primary antiviral mechanism. Nevertheless, these data expand the capabilities of heme and similar tetrapyrroles to promote beneficial effects for the innate immune system.

### Human immunodeficiency virus

Human Immunodeficiency virus is a human pathogenic RNA retrovirus of the genus *Lentivirus* first identified in the early 1980s (Barresinoussi et al., 1983; Popovic et al., 1984) and found worldwide. HIV is not a hepatotrophic virus, however as noted previously, a high percentage of patients with HIV are co-infected with HCV and/or HBV because they share common risk factors for transmission (Quan et al., 1993; Zylberberg and Pol, 1996). HIV co-infected patients, especially those with markedly depressed CD4 T cell levels and AIDS, are known to have more aggressive liver disease with increased incidence of cirrhosis as compared to patients with controlled HIV or HCV/HBV monoinfection (Telfer et al., 1994; Darby et al., 1997). Because of the global impact of the AIDS pandemic in the 1980s a tremendous research effort has been invested in drug discovery for an ever expanding list of HIV targets. While mechanistic studies of HIV are not directly applicable to the hepatotrophic viruses *per se*, the large research effort that developed model systems for anti-HIV drug design forged a path that has facilitated drug discovery for hepatitis viruses.

The antiviral interactions of heme with HIV were investigated early after the virus was discovered. These experiments established that heme has the ability to inhibit reverse transcriptases (RT) such as Rauschermurine leukemia virus RT (Tsutsui and Mueller, 1987). Ensuing work (Levere et al., 1991) showed that heme administered alone or together with 3′-azido-3′-deoxythymidine (AZT) was able to repress replication of HIV in human peripheral blood lymphocytes and H9 cell lines. This activity was found for both AZT-resistant and drug-sensitive HIV strains. Dose response studies showed significant antiviral activity at physiological levels of heme as low as 1 μM and there was a clear, enhancing effect of heme in combination with AZT. Additional work from the same group demonstrated that heme was a non-competitive inhibitor of HIV-1 RT in contrast to classical competitive inhibition with deoxythymidine triphosphates (Staudinger et al., 1996). Some early structure-function relationships were also established from testing of heme analogs and other more diverse MP structures. Interestingly, the protoporphyrin structure was found to be crucial for anti-RT activity since metal meso derivatives were inactive.

Argyris et al. (1999) then identified a unique non-nucleoside inhibitory binding site for heme and putatively other MPs located in the connection domain (region 398–407 aa residues) of HIV-1 and 2 RT. The site was revealed through heme binding assays matched to a phage-displayed 12 mer peptide library. Analysis of the peptides that actively bound heme showed that, as a group, they tended to be enriched in the aromatic amino acids Trp and Tyr while Cys residues were completely absent. There was synergism for RT inhibition with the unique heme binding site and another well-characterized non-nucleoside binding site located in the p66 palm sub domain (BHAP; Argyris et al., 1999). The authors speculated that the Tyr and Trp residues in this site of the enzyme facilitate heme binding through aromatic stacking with the planar porphyrin ring, while the propionyl and vinyl side chains anchor the holo heme.

The linear tetrapyrrole oxidation product of heme, biliverdin (BV), and its reduced derivative, bilirubin (BR) have also been reported to directly inhibit the HIV aspartic acid activated protease (McPhee et al., 1996). Using assays of recombinant HIV-1 protease, BV and BR showed nearly equal *K*_i_ values of about 1 μM for HIV-1, 2, and simian immunodeficiency virus proteases. Related tetrapyrroles showed some antiprotease activity but at much higher concentrations. It should be emphasized that the inhibitory activities of BR and BV were noted to take place at near physiological concentrations of BR (17 μM is 1.0 mg/dl). Additionally, several synthetic boronated porphyrins also showed competitive inhibition of the HIV protease with *K*_i_’s in the mid nanomolar range (Decamp et al., 1992).

## Anti-HIV Effects of HO-1 Induction on HIV

More recent work on the effects of heme on HIV replication demonstrated that heme also elicits antiviral activity through induction of HO-1 (Devadas and Dhawan, 2006). Heme was antiviral *in vitro* in a dosage dependent manner and elicited more than a 90% reduction of intracellular HIV in monocytes or T cells and was even active on drug resistant strains. While the heme antiviral targets that enabled these data were not specifically pursued, we can assume that heme likely worked through inhibition of viral uptake, RT inhibition, and/or inhibition of the HIV protease through heme oxidation products BV and BR as discussed above. An important point of this study is that it established that heme can attenuate viral levels *in vivo*. Intraperitoneal administration of heme attenuated HIV replication in humanized non-obese diabetic SCID mice carrying infected human PBMCs (Devadas and Dhawan, 2006).

### Hepatitis B virus

Hepatitis B virus is the most common chronic viral pathogen worldwide. The virus is a member of the hepadnavirus family and is one of the smallest known pathogenic human DNA viruses of about 3.2 kb. The virus replicates through a unique pre-genomic (pg) mRNA intermediate made by host nuclear enzymes which is then reverse transcribed into genomic DNA using a unique hepadnaviral reverse transcriptase. The reverse transcriptase of HBV shares a number of active site and substrate features with the HIV RT resulting in inhibitory drug overlap with HIV. However, hepadnaviral DNA synthesis employs a unique protein priming method for initiation of replication from pg RNA. The RT protein acts as both a protein primer and polymerase through interaction with a viral RNA structure (60 nucleotides) called ∈ at the 5′ end of pg RNA. This complex facilitates origin of reverse transcription, packaging, and replication fidelity (Pollack and Ganem, 1994; Tavis et al., 1994; Wang et al., 1994). In contrast to both HIV and HCV, replication and assembly of HBV occurs without a viral protease.

As compared to HIV and HCV, interactions of heme and heme oxygenase-1 with HBV have not been extensively evaluated. Nevertheless, HO-1 appears to be upregulated in response to chronic HBV infection as shown by immunohistochemical labeling of human liver samples (Abdalla et al., 2004). In a transgenic mouse model of acute and chronic HBV infection, Protzer et al. (2007) showed that induction of HO-1 attenuated viral replication as determined by reduced levels of HBV core protein. In addition to virucidal activity, HO-1 induction also led to reduced liver injury and hepato protective effects. The authors suggested that HO-1 induction worked posttranscriptionally to reduce the stability of HBV core protein (Protzer et al., 2007). Furthermore the combination of hepato protective effects from cellular injury and virucidal capabilities of HO-1 induction suggest additional benefits for potential antiviral drugs that could be designed using this important pathway.

Because heme was shown to have a specific inhibitory binding site on the HIV RT, we might predict that a similar interaction would occur with HBV. While recent evidence indicates that HBV replication is indeed inhibited by heme *in vitro*, at least one mechanism is decidedly different (Lin and Hu, 2008). Heme blocked the interaction of the RT protein with the ∈ segment of pg RNA, thus inhibiting the protein priming step of HBV replication. The heme binding site was localized to the N-terminal domain of the RT protein which is unique to hepadnaviral RT. In addition to heme, protoporphyrin free base and biliverdin were also active. While further studies of viral resistance, ligand specificity, and mechanism of these interactions need to be explored, these data suggest that similar compounds could be employed as either primary or adjunct therapy with nucleoside analogs for patients with chronic HBV infection.

## Final Considerations

In spite of development of new and improved antiviral agents for HIV, HCV and HBV, there is a persistent and urgent need for new treatment modalities as well as combination therapies. Nearly all compounds developed against these pathogens have associated problems with toxicity, viral resistance, and selectivity (De Clercq, 2007; Firpi and Nelson, 2007; Zoulim, 2011) which makes ongoing discovery of new antivirals with improved clinical spectrum and versatility necessary.

Table 1 summarizes the various sites of antiviral activity of the precursors and products of the heme oxygenase-1 system for all three viruses. It is apparent that the viral targets for heme are quite similar among the viruses in spite of viral diversity. All three viruses are inhibited by HO-1 induction and heme targets both HBV and HIV RT. Interestingly, however, heme RT inhibition occurs at different relative sites on the respective enzymes. Furthermore, heme, BV and BR inhibit both HIV and HCV proteases, in spite of the fact that the proteases differ markedly in primary structure, catalytic sites, and reaction mechanisms (Wlodawer et al., 1989; Love et al., 1996; Yan et al., 1998; Brik and Wong, 2003). Finally, HO-1 induction offers protection from oxidative injury in HBV and HCV infection thus suggesting additional benefits against oxidative induced tissue injury that is considered to be a primary chronic disease mechanism leading to hepatic injury and fibrosis (Lieber, 1997; Lee et al., 2004; Qadri et al., 2004; Seronello et al., 2007).

**Table 1:** **Activities of heme and related agents for HCV, HBV, and HIV**.

Virus	Agent	Mechanism studied	Reference
HCV	BV	Anti-NS3/4A protease	Zhu et al. (2010a)
	BV	Type I interferon induction	Lehmann et al. (2010)
	Heme	Anti-NS3/4A protease	Zhu et al. (2010a)
	Heme	HO-1 induction	Shan et al. (2007), Zhu et al. (2008)
	ZnMP	HO-1 induction and Bach 1 inhibition	Hou et al. (2008)
	ZnMP	Ubiquitination of NS5A	Hou et al. (2010)
	Fe	Antipolymerase	Fillebeen et al. (2005)
	Fe	Decreased HCV replication	Yuasa et al. (2006), Zhu et al. (2010a)
	Fe	HO-1 induction	Hou et al. (2009)
	Zn	Decreased viral replication	Yuasa et al. (2006)
	HO-1 enzyme	Enzyme overexpression	Zhu et al. (2008)
HBV	Heme	Anti-reverse transcriptase	Lin and Hu (2008)
		HO-1 induction	Protzer et al. (2007)
HIV	BV/BR	Anti-HIV protease	McPhee et al. (1996)
	Synthetic porphyrins	Anti-HIV protease	Decamp et al. (1992)
	Heme/MPs	Anti-reverse transcriptase	Levere et al. (1991), Staudinger et al. (1996), Argyris et al. (1999)
	HO-1 induction	HO-1 induction	Devadas and Dhawan (2006)
	MPs	Gp120 inhibition	Song et al. (1997)

Collectively, these findings indicate that heme and related porphyrins clearly should be considered for further *in vivo* translational studies, especially in suitable animal models and patient pilot trials, at least for proof-of-concept. The ability to inhibit multiple viral target sites on different viruses indicates that these compounds would be also useful for patients with dual or even triple infections. Co-infected patients are a serious problem worldwide and invariably present with more severe medical disease, aggressive hepatitis, and a number of treatment dilemmas (den Brinker et al., 2000; Koziel and Peters, 2007; Zhou et al., 2011). Considering the antiviral fidelity of heme and products of the heme oxygenase system it is an open question as to the why these agents have not been developed further for antiviral therapy. Heme is proven to be efficacious for use *in vivo* for acute porphyria and is usually well tolerated. Further *in vivo* studies should focus on evaluation of porphyrin based antivirals with oral bioavailability, safety, and attractive pharmacodynamic capabilities to expand their use for HCV, HIV, and HBV infections.

## Conflict of Interest Statement

The authors declare that the research was conducted in the absence of any commercial or financial relationships that could be construed as a potential conflict of interest.

## Abbreviations

BR, bilirubin; BV, biliverdin; BVR, biliverdin reductase; FL, full length; HBV, hepatitis B virus; HCV, hepatitis C virus; HIV, human immunodeficiency virus; HO, heme oxygenase; MP, metalloporphyrin; MPP, metalloprotoporphyrin; NS, non-structural; RT-PCR, reverse transcriptase polymerase chain reaction.
